# Resting-State fMRI findings in patients with first-Episode idiopathic epilepsy before and after treatment

**DOI:** 10.17712/nsj.2017.4.20160650

**Published:** 2017-10

**Authors:** Peng-Fei Qiao, Guang-Ming Niu

**Affiliations:** *From the Department of Magnetic Resonance Imaging, Affiliated hospital of Inner Mongolia Medical University, Hohhot, Inner Mongolia Autonomous Region, People’s Republic of China*

## Abstract

**Objectives:**

To detect resting-state functional MRI (rsfMRI) changes and their relationships with the clinical curative effect of anti-epileptic drugs (AEDs) for complex partial seizures (CPS) in epilepsy patients using the fractional amplitude of low frequency fluctuation (fALFF).

**Methods:**

rfMRI data from 14 CPS patients enrolled between June 2015 and June 2016 in Department of Neurology, Affiliated Hospital of Inner Mongolia Medical University were retrospectively investigated and compared with findings from 14 healthy age-, gender-, handedness-, and education-matched subjects. The patients were treated with first-line antiepileptic drugs for 12 weeks before undergoing a second rfMRI scan. fALFF data were processed using REST and SPM8 software. Whole-brain data were compared between the 2 groups.

**Results:**

The abnormal findings in CPS patients significantly decreased or disappeared after AED treatment.

**Conclusion:**

Measuring fALFF is useful for identifying brain functional changes in patients with first-episode epilepsy before and after treatment. Performing rfMRI is useful for evaluating treatment responses and may provide new insight into the pathophysiological mechanisms of epilepsy.

Idiopathic epilepsy is a chronic neurological disease characterized by no organic or metabolic brain abnormalities, varying degrees of autonomic symptoms, consciousness disturbance, and psychiatric symptoms. It may be related to genetic factors, but the pathogenesis is not fully understood.^1^ Patients with this condition experience sudden, transient and recurrent dysfunction of central nervous system caused by super-synchronous abnormal discharges of brain neurons.^2^ Idiopathic epilepsy has complex physiological mechanisms and diverse clinical manifestations. Medication is the primary and most commonly used treatment. Assessing drug efficacy and the development of neurological lesions by non-invasive magnetic resonance imaging (MRI) provides objective evidence for new drug development and clinical treatment.^3^ In this study, fractional amplitude of low frequency fluctuations (fALFF) was measured using resting-state functional MRI (rfMRI) to investigate changes before and after treatment and their relationship with the clinical efficacy of antiepileptic drugs (AEDs).

## Methods

This was a retrospective study of 14 patients diagnosed with primary complex partial seizures (CPS) between June 2015 and June 2016 in the Department of Neurology, Affiliated Hospital of Inner Mongolia Medical University. The cohort included 8 males (9-35 years, mean 22.4 years) and 6 females (8-40 years, mean 25.2 years). Fourteen healthy volunteers matched with the patient group in age, gender, handedness, and years of education were selected as controls. This study was approved by the Medical Ethics Committee of Inner Mongolia Medical University Affiliated Hospital. All subjects signed an informed consent form.

Typical primary CPS was diagnosed according to the 2007 diagnostic criteria of the International League Against Epilepsy (ILAE). Only first-episode patients were enrolled to avoid the influences of disease course and drugs. All patients were asked on their detailed medical history by a clinically experienced deputy chief physician or another more highly placed neurological professional. Seizure type was determined based on medical history and electroencephalography (EEG) changes. Neurological examinations were performed to exclude serious physical diseases and complications, and conventional brain MRI scans were taken to exclude apparent structural abnormalities.

The rfMRI was performed in both the patient and control groups using a GE Discovery 750 3.0T superconductive MRI scanner (GE Healthcare, Chicago, IL, USA). A second scan was performed in the patient group after 12 weeks of treatment with the conventional first-line AED carbamazepine.

Subjects laid awake on the table with noise-blocking headphones and were instructed to close their eyes, keep their heads still, and try not to think or perform specific cognitive tasks. Scanning did not begin until subjects adapted to the environment.

Functional scanning was performed using echo planar imaging with gradient recalled echo sequences (field of view 24 cm×24 cm, 64×64 matrix, flip angle 90°, repetition time 2000 ms, echo time 30 ms) with whole-brain coverage using 28 oblique axial 4-mm slices with 4-mm spaces. The first 10 images were excluded owing to T1 equilibrium effects. Resting-state scanning lasted for 512 s, producing 256 brain volume data sets. rfMRI data of the 256 volumes of each subject were used for analysis. Data preprocessing was performed using the SPM8 software package (statistical parametric mapping, http://www.fil.ion.ucl.ac.uk/spm/). After slice timing, realignment, normalization, smoothing, and filtering, the time series of each voxel was transformed to the frequency domain to obtain the power spectrum. Next, we calculated the square root at each frequency of the power spectrum to obtain the averaged square root across 0.01–0.08 Hz at each voxel. This averaged square root was taken as the ALFF, and fALFF is calculated as the ratio of ALFF between 0.01 and 0.08 Hz to the ALFF over the entire frequency range. Lastly, the statistical parametric brain maps of the amplitude were obtained to describe spontaneous activity of voxel intensity. The MATLAB-based resting-state data analysis tool REST (www. restfmri.net) was used to assess the fALFF maps. Voxelwise two-sample t-tests were employed to identify the increased or decreased activity regions of CPS patients compared to healthy controls before and after treatment. The t-value was set to *p*<0.05 after Alphasim correlation.

## Results

Compared with the control group, the untreated patient group showed significantly higher fALFF in the bilateral thalami, posterior cingulate, left angular gyrus, and inferior parietal lobule and significantly reduced fALFF in the bilateral medial prefrontal lobes and anterior cingulate. After treatment, abnormally activated areas decreased or disappeared in the thalamus and angular gyrus and increased in the left inferior parietal lobule. In addition, newly activated brain areas were detected in the bilateral frontal lobes, precuneus, and left cuneus (**Tables 1, 2, and 3**).

**Table 1:** Regions showing increased fALFF between healthy subjects and untreated epilepsy patients.

Regions	L/R	MNI (X, Y, Z)	Voxels (mm^3^)	t-Score
Thalamus	R	14, -30, 10	28	2.9962
Thalamus	L	-12, -28, 9	35	3.6621
Posterior cingulate	R	-13, -50, 16	31	3.5521
Posterior cingulate	L	12, -45, 18	28	2.7536
Angular gyrus	L	-56, -47, 39	35	3.8926
Inferior parietal lobule	L	-50, -27, 25	28	3.6249

R - right, L - left, MIN - Montreal Neurological Institute coordinate, fALFF -fractional amplitude of low frequency fluctuation

**Table 2:** Regions showing decreased fALFF between healthy subjects and untreated epilepsy patients.

Regions	L/R	MNI (X,Y,Z)	Voxels (mm^3^)	t-Score
Medial prefrontal lobe	R	3, 55, 25	26	-2.7786
Medial prefrontal lobe	L	-7, 63, 15	38	-3.6603
Anterior cingulate	R	3, 9, 24	19	-2.9482
Anterior cingulate	L	-7, 15, 26	21	-3.1325

R - right, L - left, MIN - Montreal Neurological Institute coordinate, fALFF -fractional amplitude of low frequency fluctuation

**Table 3:** Regions showing increased fALFF between healthy subjects and treated epilepsy patients .

Regions	L/R	MNI (X, Y, Z)	Voxels (mm^3^)	t-Score
Angular gyrus	L	-54, -45, 37	13	2.1689
Inferior parietal lobule	L	-55, -23, 27	38	3.6649
Frontal lobe	L	-42, 30, 15	20	2.0922
Frontal lobe	R	40, 33, 19	23	2.6839
Precuneus	L	-7, -43, 46	26	3.5513
Precuneus	R	9, -52, 35	20	3.0015
Cuneus	L	-8, -61, 53	35	2.5427

R - right, L - left,

## Discussion

Low-frequency amplitude analysis was first developed and applied to resting-state fMRI research by Zang et al.^4^ It reflects the amplitude of blood-oxygen-level dependent signal changes from baseline. Data between 0.01 and 0.08 Hz were included in the analysis. Spontaneous activity of brain regions in the resting state is reflected from the perspective of energy, and neuronal excitability and metabolism are measured by ALFF^5^ to identify functional changes in the brains of patients with epilepsy. However, ALFF is sensitive to physiological noise. To this end, Zou et al.^6^ proposed the use of fALFF. This approach effectively inhibits interference from the large vascular compartment and ventricular system, improving the specificity of detecting spontaneous brain activity and better reflecting functional brain characteristics.

The results of this study show that compared with the control group, patients with untreated CPS exhibited significantly higher fALFF in the bilateral thalami, posterior cingulate, left angular gyrus, and inferior parietal lobule and significantly reduced fALFF in the bilateral medial prefrontal lobes and anterior cingulate. These findings are consistent with those of previous studies.^7^ These regions were distributed in a roughly symmetrical pattern. Some inconsistencies in affected brain regions may be related to differences in age, gender, and research methods. The observation of abnormal brain activity over a wide area once again demonstrates that seizure activity begins and spreads based on abnormalities in an extensive neural network.

The thalamus is the brain’s most important sensory conduction and relay station and plays a role in the initial facilitation and spread of seizure activity.^8^,^9^ It is composed of several gray nuclei, each with a different role. It is most strongly implicated in propagating partial seizures.The posterior cingulate is mainly involved in regulating brain activity related to memory,^10^ but it is also an important part of the intracranial spreading network of seizure activity. Metabolic activity in the posterior cingulate increases with seizure activity. This affects its own functioning, which, in turn, explains the confusion and blackouts experienced during seizures and the subsequent development into amnesia and other mental disorders over time. The angular gyrus is the human visual language center, also known as the reading center.^11^ Damage to this area is presumably associated with cognitive language dysfunction in epilepsy.^12^ In this study, these brain regions were significantly activated with increased neuronal excitability before AED treatment. We believe that the regulation and spread of epileptiform discharges are directly related to dysfunction of these brain areas. However, activation in these areas was significantly reduced or disappeared after treatment, suggesting that the abnormalities normalized. Zhang et al^13^ analyzed EEG characteristics in children with benign epilepsy and their correlations with cognitive level before and after treatment. They reported significantly reduced discharges and significantly improved EEG findings after treatment. The findings of Zhang et al^13^ and the present study suggest that AED treatment can substantially reduce abnormal brain activity in patients with seizures.

Importantly, activity in some small areas was not significantly affected by treatment. The activated area in the left inferior parietal lobule actually became larger. Damage to the inferior parietal lobule is related to limited memory capacity. Zhang et al^13^ reported that patients with inferior parietal lobe damage had obvious memory impairment and a significantly lower memory quotient than the control group. There may be 2 reasons for persistent and worsened abnormalities. Firstly, prolonged seizures cause hypoxia and consequent irreversible brain damage.This could be a characteristic of idiopathic epilepsy that serves as the neural basis of seizure recurrence. Secondly, it may be related to the effects of long-term medication. The pharmacological mechanism requires further investigation.

Patients with untreated CPS had significantly reduced fALFF in the bilateral medial prefrontal lobes and anterior cingulate compared with the control group. These abnormalities reduced or disappeared after treatment. Activity in the bilateral medial prefrontal lobes and anterior cingulate was inhibited before treatment, which resolved after 12 weeks of AED use. However, newly activated brain areas included the bilateral frontal lobes, precuneus, and left cuneus. These regions belong to or are near the “default mode network” (DMN).^14^ This suggests that as epileptic activity initiates and spreads, leading to abnormal brain activity, the DMN is inhibited, resulting in negative activation of the network. When the network can maintain high activity after treatment, this manifests as normal activation at rest. These results further support the hypothesis that AEDs decrease or reverse epileptic activity in specific brain regions.

Follow-up assessments confirmed that patients had no or significantly decreased seizures after treatment. This suggests that AEDs partially reversed abnormal brain function while improving the symptoms. Jansen et al^15^ used fMRI to examine the effects of topiramate on abnormal brain activity in patients with cognitive language impairment due to epilepsy. They found obvious activation of the prefrontal lobe in the treatment group compared with the control group, indicating that the drug normalized brain function, which is consistent with the findings of the present study. It should be noted that the sample size was small in this study. As a result, stratified analysis by age, gender, education level, and other factors was not performed. The results therefore require further verification in more subjects. Although fALFF data can be obtained from the whole brain, functional characteristics of specific brain regions might be neglected with this approach.

In conclusion, there are few comparative rfMRI studies on brain network abnormalities before and after epilepsy treatment. To avoid the influence of medication and disease course, only first-episode patients were enrolled in this study. fALFF data of the whole brain were analyzed and compared. These are innovations of the present study. In this way, the changes in brain function before and after treatment can be better revealed, and the treatment response in epilepsy and efficacy of antiepileptic drugs can be objectively assessed, thus contributing to further research of the complex pathophysiology of epilepsy. Therefore, the sample size needs to be increased in future studies, and multimodal functional imaging should be combined with fALFF to improve result credibility and support clinical and research efforts.
